# Erythropoietin use in the newborn

**DOI:** 10.1186/1824-7288-40-S2-A41

**Published:** 2014-10-09

**Authors:** Giuseppe Buonocore

**Affiliations:** 1 Department of Molecular and Development Medicine, University of Siena, 53100 Siena, Italy

## 

The first descriptions of blood transfusion in neonates date back to the nineteenth century. Time is past but uncertainties still remain considerable on optimal red blood cell (RBC) transfusion use in an era of evidence-based medicine. The need for RBC transfusion in critically ill neonates is differently judged among clinicians due to a lack of an agreement on measures of the need for transfusion. However some studies have shown an association between preterm infant morbidities, such as necrotizing enterocolitis and significant intracranial hemorrhage, with transfusions. The erythropoietin (EPO) use might prove clinically important. Physiologically in all newborns there is a progressive fall in hemoglobin concentrations to the nadir at about 8–12 weeks of life. The nadir is even lower and sooner in preterm neonates. This anemia of prematurity is the consequence of multiple factors, some are physiological processes as vulnerability of red cells to oxidative damage, reduced red cell lifespan when compared to the adult, lower erythropoietin (EPO) response to anemia than in older ages, increased requirements due to the postnatal growth; some are non-physiological processes as iatrogenic blood sampling, inter-current illnesses and sepsis. All these factors are responsible for low plasma EPO concentration in preterm newborns. This provides a rationale for the use of EPO in prophylaxis or treatment of the anemia. EPO has been used clinically for more than 20 years and many randomized clinical trials demonstrated that EPO successfully stimulate erythropoiesis and decrease the need of transfusion in anemic adults and children with end-stage renal disease or cancer. Differently in preterm newborns, the EPO treatment has demonstrated varied success in decreasing the total number and volume of transfusions. There are not clear evidences from high quality trials to define the absolute requirement and benefit for neonatal RBC transfusion. A recent Cochrane metanalysis performed by Ohlsson A. and Aher S.M. (1) to assess the effectiveness and safety of early initiation of EPO or darbepoetin in reducing RBC transfusions in preterm and/or low birth weight infants, concludes that the small reductions in RBC transfusion observed after EPO treatment are of limited clinical importance. In contrast the possibly increased risk of ROP, does not recommend the administration of EPO. Darbepoetin requires further study for definitive conclusions.
